# Discovery as public infrastructure: Renewing the social contract for science

**DOI:** 10.1093/pnasnexus/pgag312

**Published:** 2026-10-13

**Authors:** Sergio Carbajo, Asad Madni, Julia Powles

**Affiliations:** Department of Electrical and Computer Engineering, University of California, Los Angeles, CA 90045, USA; Department of Physics and Astronomy, University of California, Los Angeles, CA 90045, USA; SLAC National Accelerator Laboratory, Stanford University, Menlo Park, CA 94025, USA; California NanoSystems Institute, Los Angeles, CA 90095, USA; Department of Electrical and Computer Engineering, University of California, Los Angeles, CA 90045, USA; Institute for Technology, Law & Policy, University of California, Los Angeles, CA 90045, USA; School of Law, University of California, Los Angeles, CA 90045, USA; Sydney Law School, University of Sydney, Sydney, New South Wales 2050, Australia; Centre for Business Research, Judge Business School, University of Cambridge, Cambridge CB2 1QA, United Kingdom

## Abstract

Scientific capacity built over generations is being weakened by abrupt political disruption just as discovery is becoming faster, more convergent, globally networked, and more socially consequential. Restoring funding and protecting scientific institutions are urgent, but not sufficient: the postwar compact that sustained discovery before its uses were known must be renewed for 21st-century conditions and made resilient to arbitrary disruption. We propose the Discovery Compact, a renewed social contract among government, science, industry, and the public that treats discovery as shared public infrastructure—capacity a plural society builds, tends, and holds in common across generations. It is organized around five criteria: (i) sustaining discovery as national infrastructure; (ii) enabling institutions to move at speed without sacrificing rigor; (iii) building scientific capacity through people, openness, and mobility; (iv) translating discovery without depleting its source; and (v) securing democratic legitimacy and institutional resilience. Together, these criteria direct policy toward durable scientific capacity and insulate against indiscriminate deregulation, compulsory commercialization, arbitrary institutional disruption, and political control of scientific judgment. This Perspective is not a legislative template but an invitation to a national and international process of protecting and renewing the institutions and infrastructure through which societies discover, generate, protect, and prepare.

## The compact that built the modern world

The postwar compact for science created extraordinary national capability by sustaining discovery before its uses were known. That capacity is now being weakened by abrupt political disruption just as research is becoming faster, more convergent, globally networked, and socially consequential. The United States must therefore restore and protect its scientific institutions while renewing the compact that governs them for 21st century conditions. The most consequential investments in science are often made before their uses can be named. Vaccines, semiconductor chips, satellite navigation, weather forecasting systems, medical imaging and treatments, digital communications, modern agriculture, and the tools now accelerating AI all emerged from institutions willing to support uncertainty over long periods—the same lineage the public already recognizes in the internet, GPS, and the mRNA vaccines developed at speed during the COVID-19 pandemic. Their origins were rarely linear. They depended on basic research, engineering, instrumentation, standards, research facilities, and generations of people trained before a market or crisis made their value obvious. Vannevar Bush’s 1945 report, Science: The Endless Frontier (1), gave this logic a durable political form. Before that, Bush had directed the wartime Office of Scientific Research and Development, which reported directly to President Roosevelt and mobilized some 6,000 scientists behind radar, the mass production of penicillin, and the Manhattan Project. As the war drew to a close, Roosevelt asked Bush how that partnership between government and science might be sustained in peacetime. Science: The Endless Frontier offered a solution and led, 5 years later, to the National Science Foundation. The report is commonly remembered as an argument for federal funding of basic research (2). But its mission was to deepen a proposition about institutional responsibility; specifically, that a democratic government should cultivate knowledge and talent whose benefits are broad, uncertain, and difficult for nongovernmental or private actors to capture. The resulting postwar compact connected federal agencies, research universities, national laboratories, mission-oriented programs, professional communities, and industry. It did not eliminate competition among those institutions, nor did it create a perfectly equitable or efficient system. It did, however, establish the expectation that discovery was a long-term public responsibility rather than a sequence of isolated projects. That expectation generated enormous public value. Research and innovation create benefits that extend far beyond the organizations that initially fund them, because knowledge spreads across firms, sectors, places, and generations. Estimates of the total benefits to society therefore consistently exceed the financial returns captured by individual investors (3). Jones and Summers (4) conclude that these broader social returns are sufficiently large to imply substantial underinvestment relative to the public optimum. Recent macroeconomic models find that public R&D can raise private productivity and stimulate additional private investment rather than replace private investment (5, 6). Newly assembled patent and funding data further suggest that government-funded innovations, although a small share of total patents, have played an outsized role in medium-term productivity growth (7). These returns are not a historical accident, and they cannot be assumed to reproduce themselves. The postwar compact worked because it sustained capabilities—laboratories, facilities, measurement systems, clinical networks, open scientific communities, and talent pipelines—before they became indispensable. Yet these institutions are often treated as if they could be switched off and on without lasting cost. A laboratory may be closed in weeks but take years to rebuild; a cohort of students or postdoctoral researchers may redirect its ambitions in a single funding cycle, while the lost expertise takes a decade to replace; clinical, manufacturing, or data infrastructure interrupted today may reveal its absence only later, when society reaches for capability that was never sustained. The question is not whether the postwar compact succeeded. It inarguably did. The question is whether the institutional architecture that produced that success is sufficient for the century now unfolding. We argue it is not. We focus on the United States because it is where the postwar compact was forged, and because the scale and international reach of the American research system make its destabilization consequential far beyond national borders. Our argument joins a crowded and urgent conversation about how to save American science but departs from it in scope. One strand responds to the present crisis by calling for a renewed consensus that recommits to Vannevar Bush’s essentials (8), i.e. federal support for basic research, cross-sector partnership, and international openness, coupled with a strategic reset around talent, funding diversification, and clearer priorities (9). A second, metascience strand takes the diagnosis that scientific progress has slowed and grown more costly and proposes mechanism-level fixes (10): portfolio funding, fast grants, risk-tolerant program officers, and independent institutes. A third, older tradition argues the postwar social contract itself is no longer sufficient (11) and that recognizing use-inspired basic research can ground a new compact between science and government (12). We build on all three. Our contribution is to treat the problem as one of institutional architecture rather than any single lever, arguing that restoration is necessary but insufficient, that discovery should be governed as national infrastructure, and, distinctively, that accountability must run in both directions: scientists and research institutions must remain answerable for integrity, responsible stewardship, and public value, while public decision-makers must be answerable for the scientific capacity their choices preserve or destroy.

## Restoration is necessary but not sufficient

Restoring funding and prior institutional arrangements is urgent but far from enough, because the postwar system now faces five structural mismatches—in speed, convergence, talent, institutional resilience, and legitimacy—between the assumptions on which it was built and the conditions under which discovery now occurs. None of these mismatches can be closed by funding alone. The first mismatch concerns speed. In many emerging domains, competitive and scientific landscapes change over months rather than decades. AI models, automated experimentation, biotechnology platforms, advanced materials, quantum systems, and semiconductor technologies can develop faster than conventional proposal, review, appropriation, procurement, and regulatory cycles. Traditional peer review remains indispensable for assessing quality and allocating public funds. Yet it is not equally well suited to every class of scientific opportunity. Empirical work indicates that highly novel proposals often produce greater disagreement among reviewers, making them less likely to achieve the consensus scores on which many funding systems rely (13). A system designed primarily to minimize failure can also minimize the probability of transformational success. The second mismatch is convergence. Much of the 20th-century research system was organized around comparatively stable disciplinary and agency boundaries. Today’s most consequential problems and opportunities increasingly refuse those categories. Consider the protein-folding problem. For more than 50 years, predicting a protein’s 3D structure from its amino-acid sequence resisted solution (14). In 2020, DeepMind’s AlphaFold2 predicted structures with accuracy competitive with experiment, effectively solving it, but not through machine learning alone. The breakthrough rested on the Protein Data Bank, a public-good archive established in 1971 and sustained for half a century, whose corpus of more than 230,000 experimentally determined structures (15) was the training data without which no algorithm could have reached constitutive success; open-access structural data were itself the cornerstone of the AI advance (15). Computer science, decades of structural biology, and an openly shared public-data infrastructure converged to solve a problem that none could have cracked apart. The point is not confined to AI. The mRNA COVID-19 vaccines reached authorization in under a year, not by luck but because decades of federally supported basic research—in RNA biochemistry, immunology, and nucleoside modification—had already assembled the components, which a public–private effort could then integrate with genomics, manufacturing, clinical trials, and regulatory review at emergency speed (16). No single discipline or agency produced that capability; the response was fast only as a result of pre-existing convergent infrastructure and talent. AI models are rapidly entering pharmaceutical research and other discovery-intensive fields (17). The relevant policy targets are therefore not always individual disciplines or agencies, but shared infrastructures, methods, and knowledges, and people capable of working across domains and institutions. The third mismatch concerns human talent. Scientific systems compete through talent and networks at least as much as through budgets. Yet the early-career pipeline has become longer, less predictable, and more precarious. A longitudinal study spanning 38 countries and 16 disciplines found that roughly one-third of researchers stopped publishing within 5 years of beginning their careers and about half within 10 years, with attrition varying across fields and cohorts (18). Attrition is not confined to the earliest years. A census of roughly 245,000 US tenure-track and tenured faculty found that women leave academia at higher rates than men at every career stage, including among the already tenured, and are more likely to feel pushed out by workplace climate than pulled toward better opportunities (19). Much of this loss is structural rather than a matter of individual choice: parenthood alone accounts for a sizeable portion of the gender productivity gap, setting mothers back roughly 5 years relative to comparable colleagues (20). A system that keeps shedding experienced women forfeits exactly the mid-career expertise that, as noted above, takes a decade or more to rebuild. At the same time, the United States has historically converted international mobility into domestic scientific and technological capacity. High-skilled foreign talent accounts for a disproportionate share of American invention, including spillovers that increase the productivity of collaborators (21). Historical restrictions on scientific immigration also offer a cautionary lesson: when access is narrowed, the loss is not only to individual migrants but to the networks of invention that would otherwise form around them (22). The fourth mismatch is institutional fragility. Research unfolds over long horizons, while budgets, advisory structures, regulations, and political priorities can change abruptly. Disruptions in the 2020s have made that asymmetry unusually visible, including the removal of the National Science Foundation’s governing board and major uncertainty around agency budgets (23). These events matter beyond any one administration. They expose a structural vulnerability: scientific institutions can be destabilized much faster than scientific capacity can adapt. Political leadership must retain the authority to set priorities and scrutinize public spending, but democratic mechanisms should not come at the cost of continuous vulnerability for expert judgment, institutional memory, and long-term research programs. Accountability, moreover, cannot run in only one direction. The research enterprise is dense with mechanisms that hold scientists answerable (24, 25)—for spending, safety, integrity, and results—yet few comparable mechanisms hold public decision-makers answerable for the scientific capacity their choices preserve or destroy. When a governing board can be dissolved, or a long-term program terminated with no assessment of the capability lost (26), the burden of disruption falls on the public at large, especially on the most vulnerable populations. A renewed compact should therefore make the stewardship of national scientific capacity an explicit object of public accountability, so that abrupt destabilization carries clearly articulated cost and accountability measures.

Administrative burden creates a quieter, yet equally corrosive form of fragility. Researchers have repeatedly reported that a substantial share of federally funded effort is consumed by proposal preparation, compliance, reporting, and overlapping—sometimes redundant—institutional requirements (27). Some requirements are essential to safety, integrity, and accountability. The cumulative system, however, often measures procedural compliance more readily than the outcomes those procedures are intended to protect. The National Academies have called for a more coherent and risk-proportionate regulatory framework for academic research (28). Reclaiming researchers’ time is not administrative housekeeping; it is a direct action to increase the national research capacity. The compact faces a legitimacy mismatch. Public confidence in science remains stronger than confidence in many institutions, but it has declined and become more politically differentiated (29). The scientific community often responds by emphasizing public education and communication, but legitimacy cannot be produced by communication alone. It depends on whether institutions are visibly accountable, whether benefits are broadly and tangibly shared, whether communities can participate in decisions that affect them, and whether scientific independence is paired with public responsibility (30, 31). Finally, restored investment must be accompanied by safeguards against arbitrary disruption, because scientific capacity takes years to build and can be destroyed in a single political cycle. These mismatches point toward a conclusion that is easy to postpone but difficult to avoid. The United States does not simply need to repair and refinance a research system whose capacity is being eroded. It needs to renew its social contract, the Compact that defines why the system exists, who is responsible and accountable for sustaining it, and how it should operate under 21st-century conditions.

## Defining the discovery compact

We deliberately set aside the metaphor of the “Endless Frontier” and name our subject plainly: a renewed social contract for science. A compact is not a single agency, funding bill, or centralized plan for scientific priorities. Nor is it a defense of every existing practice. It is an allocation of responsibilities and expectations (32). The government sustains capabilities whose benefits are too broad, uncertain, or delayed for private actors to support alone. Research institutions protect rigor, openness, integrity, and the conditions for intellectual risk. Industry, mission agencies, philanthropy, and public institutions build pathways through which knowledge becomes useful. Researchers accept responsibility not only for excellence but also for the social and institutional contexts into which their work enters (33). Democratic institutions provide oversight while protecting the independence of scientific assessment. The public, in its complex and rich plurality, is not merely an audience for completed discoveries but a participant in defining legitimate purposes, acceptable risks, and shared value (34). Our organizing metaphor for the compact is infrastructure. Discovery is upstream public infrastructure: the capacity to generate knowledge, train people, operate facilities, maintain datasets and standards, and respond to needs that cannot be fully predicted in advance. The analogy does not imply that science should become rigid, centrally directed, or insulated from evaluation. It means that the capability to discover must exist before demand becomes urgent. Research policy is, in this sense, disaster policy written in advance. The absence of preparedness often becomes visible only when a crisis exposes the experiments, expertise, supply chains, clinical networks, or measurement systems that were never built. The Discovery Compact, whose tenets are illustrated in Fig. 1, rests on five design criteria: (i) sustaining discovery as infrastructure; (ii) building institutions that can move at the speed of science; (iii) building scientific capacity through people, openness, and mobility; (iv) translating discovery without depleting it; and (v) securing democratic legitimacy and institutional resilience.

**Figure 1:**
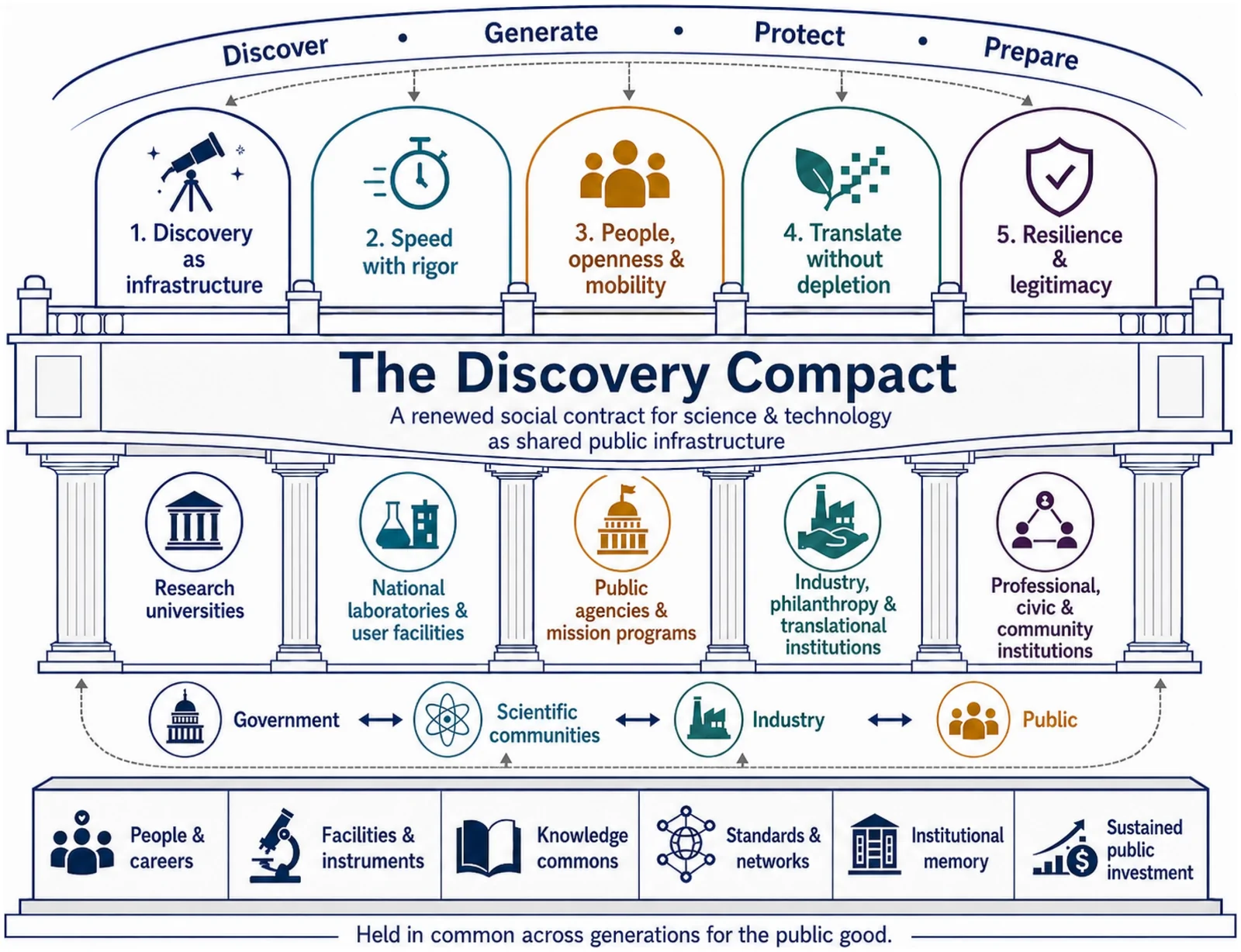
The Discovery Compact. A renewed social contract for science and technology that treats discovery as shared public infrastructure. Five design principles guide the compact: sustaining discovery capacity, enabling speed with rigor, centering people and openness, translating knowledge without depleting its source, and building institutional resilience and democratic legitimacy. These principles are supported by an institutional architecture spanning universities, national laboratories, public agencies, translational organizations, and civic and professional institutions, and by shared foundations including people, facilities, knowledge commons, standards, institutional memory, and sustained public investment.

## Treat discovery as national infrastructure

Long-horizon discovery capacity is a national asset. Public research differs structurally from ordinary procurement or near-term industrial investment. But basic research today is organized more like a marketplace of ideas than a collective, multiscale infrastructure spanning generations of people, facilities, disciplines, and institutions. Its benefits are uncertain in form, distributed, and often realized decades after the original work. These characteristics are precisely why markets underinvest and why the public role is indispensable. Yet aggregate national R&D spending can obscure a consequential shift in who supports basic research: NSF data reported by Mervis show that the federal share of US basic-research funding fell from 61% in 2004 to below 50% in 2013, and to 44% by 2015 (35). Infrastructure should be understood broadly. It includes major research facilities, but also interdisciplinary collaborations, instrumentation, shared testbeds, standards, repositories, longitudinal datasets, clinical and field networks, research software, technical staff, and institutional memory. It includes the human capability to operate these systems and to imagine uses for them that were not visible when they were created. Biomedical history illustrates the point: foundational NIH-supported research contributed across the knowledge base underlying later drug approvals, even when the connection between an early grant and an eventual therapy was neither direct nor foreseeable (36, 37). Policy should therefore be judged by whether it preserves and enlarges discovery capacity over time. Predictable multiyear support, transition planning before major disruptions, and regular assessments of national capability belong on the dartboard; abrupt decisions that ignore the time required to rebuild teams, facilities, and knowledge do not. Because these capabilities cannot be rebuilt quickly, a renewed compact should protect continuity through predictable multiyear funding, transition plans for programs and facilities facing major changes, and regular assessments of whether national research capacity is being built or lost. These safeguards would not exempt science from democratic budgeting or performance review; rather, they would require decision-makers to account explicitly for the long-term capabilities their choices preserve or dismantle.

## Build institutions that move at the speed of science

Long-term investment is insufficient if institutions cannot recognize and support important opportunities while they remain open. A modern compact must therefore reconcile rigor with speed—between a centralized, peer review-based research assessment and support system and a plural, differentiated system in which the form of review matches the type of uncertainty being evaluated. Conventional peer review should remain the primary pathway for much public research. Alongside it, agencies need protected mechanisms for cutting-edge work characterized by unusual novelty, convergence, time sensitivity, or technical risk. The relevant precedents are not perfect templates. DARPA, ARPA-E, and ARPA-H demonstrate that public institutions can grant program managers greater portfolio authority, use staged commitments, and pursue ambitious missions without abandoning accountability. These pathways should be designed to tolerate informative failure while remaining transparent about goals, conflicts, and performance. This flexibility must be sharply distinguished from “discretion” of a different kind, epitomized by the so-called proposed OMB rules (38). OMB’s proposed 2026 rule, which it aims to make effective by 2026 October 1, would recast federal grant guidance as a binding regulation, require senior appointees to review discretionary awards before issuance, make peer-review recommendations explicitly advisory, and permit agencies, to the extent allowed by law, to terminate awards that no longer advance program goals, agency priorities, or the national interest. This centralization of political control deepens the very institutional fragility that a renewed compact must reduce. Instead of the ARPA model, which delegates discretion to accountable technical experts and bounds it by mission and transparency, the OMB rules concentrate political influence and bound science by partisan alignment. At the moment, the rule’s ultimate form and implementation remain contested, and may change through ongoing legislative, administrative, or judicial action. Speed also depends on how researchers spend their time. The administrative burden of research has expanded through many individually defensible requirements, including financial controls and disclosures, export compliance, cybersecurity and research security, laboratory safety, and ethical protections for animal welfare and human research participants, together with the associated mandatory training. The problem is cumulative: requirements are often layered across agencies and institutions without a mechanism for assessing their combined cost or effectiveness, most often producing unnecessary duplication and an overload of redundancies. The compact should treat researcher time as a scarce national resource and require that new obligations be proportionate to risk, interoperable across agencies, reusable across grants and institutions where possible, and evaluated against the scientific capacity they consume (39). Even in the Federal Demonstration Partnership’s faculty survey 15 years ago (40), principal investigators reported that roughly 42% of their federally funded project time went to administration rather than research; more recent assessments find that share essentially undiminished, with researchers still spending nearly half of their research time on grant-related paperwork rather than discovery (27). This principle extends to procurement, access to facilities, and data infrastructure. Cutting-edge science increasingly depends on specialized components, cloud computing, shared instruments, and quickly developing numerical models; when administrative systems move more slowly than the technologies they govern, the delay is not neutral, changing which questions can be asked and which institutions can participate. It also carries a quieter cost. When public structures are too slow or too burdensome, researchers route their most time-sensitive work into private ones, such as the industry labs and start-ups that already dominate fields such as AI (41), trading procedural delay for weaker public oversight and deeper financial conflicts of interest, which are already widespread where academic and commercial interests meet (42). Agility, then, is not merely efficiency; it determines whether emerging work stays inside institutions accountable to the public. A policy meets this criterion when it shortens the path from idea to responsible experimentation without weakening rigor. Differentiated review, staged funding, interoperable compliance, and faster procurement belong on the dartboard; blanket deregulation and political preclearance of scientific awards do not.

## Place people, openness, and mobility at the center

The ultimate constraint on scientific capacity is human talent. Facilities, grants, and computing systems matter because skilled humans know how to use them, challenge their assumptions, and combine them in ways no central plan could specify. Yet workforce policy is too often treated as downstream of research policy, as though talent will simply appear once programs are funded (43). A renewed compact must treat people, mobility, and the scientific community as a combined, core infrastructure. That begins with early-career researchers. Graduate students, postdoctoral scholars, staff scientists, research engineers, technicians, physician-scientists, data stewards, and other emerging professionals are often closest to new tools and methodological shifts (44). They are nevertheless rarely represented in the governing or advising bodies that set institutional or national priorities. The standard response to workforce instability is to offer more fellowships or bridge funding. Those measures are important, but stabilization without voice leaves a strategic resource unused. Early-career researchers should be agenda-setters: participants in advisory structures, roadmapping exercises, program design, and the evaluation of how policies operate in practice. The compact must also preserve a calibrated openness—welcoming talent and collaboration while applying proportionate safeguards for integration, safety, and national security—through which the United States has historically attracted and multiplied talent. Skilled immigration does more than fill vacancies. It forms networks of invention, collaboration, entrepreneurship, and mentorship whose benefits spill across institutions and regions. International scientific mobility is therefore not peripheral to competitiveness; it is one of its mechanisms. Glennon shows that restrictions on skilled immigration can influence where firms locate innovative activity, because organizations may move the work rather than simply abandon it (45). Openness does not preclude safeguards for safety, integrity, and national security. Some research, data, technologies, and collaborations require protection, but broad-brush restrictions untethered from specific risks can erode the very scientific capacity they seek to secure by weakening trust, mobility, and collaboration. Evidence of declining US-China scientific exchange illustrates how rapidly networks can contract under geopolitical pressure (46). The better approach is risk-specific and capability-specific: preserve the openness of the large majority of research while creating trusted advisory structures and appropriately secure environments for the narrower domains that justify them. As Shih and Wagner argue, indiscriminate securitization can inhibit the practice of science without delivering the competitiveness gains promised in its name (47). Finally, talent policy must consider what researchers are trained to see. Advanced technologies reshape institutions, labor, access, power, and public life. Technical excellence alone does not equip scientists and engineers to reason about those consequences. Curricular breadth, including engagement with ethics, history, social science, law, and the humanities, should be treated as part of scientific capability rather than an optional supplement (48). The point here is that principled innovation is durable innovation. Researchers need to develop the skills, knowledge, and collaborations necessary to exercise judgment in the environments their work will transform. The corresponding policy aim is a scientific workforce with genuine staying power and agency: durable career pathways, a voice in institutional decisions, mobility constrained only by specific demonstrated risks, and training broad enough to support judgment about the worlds research will shape. More fellowships alone, blanket restrictions, or narrowly technical preparation fall short.

## Translate discovery without depleting it

The public value of science depends on translation, but the language of translation often collapses distinct activities into a dominant expectation of rapid commercialization. This can create a damaging inference that if research does not yield a product, company, or operational deployment on a short horizon, it has failed to produce value. The compact should reject that premise. Discovery and translation are complementary but institutionally different. Foundational research explores uncertain questions, creates methods and instruments, and trains people whose eventual contributions cannot be fully specified. Translation integrates knowledge with manufacturing, regulation, clinical evidence, systems engineering, procurement, market formation, and user needs. The valley between them is real (49), but the solution is not to pull all upstream research into downstream metrics (50). It is to build stronger bridge institutions alongside discovery.

Those bridges can take many forms, including but not limited to shared testbeds, user facilities, translational centers, mission-oriented programs, regional consortia, public–private co-development, standards organizations, agency-adjacent foundations, and challenge-driven funding. Their common function is to assemble capabilities that no single laboratory or firm can provide. They should enable researchers to move ideas toward use without strictly requiring every laboratory to become a start-up or every grant to promise a product. This distinction is especially important in fields where enabling technologies are distributed across many sectors. Photonics, advanced instrumentation, measurement science, computation, and materials research underpin communications, health, defense, semiconductor manufacturing, energy, and quantum systems simultaneously. Their public value cannot be assigned to a single application or sponsoring agency. The same is true in biomedicine, where foundational research may support entire classes of future therapies rather than one molecule. Translation policy should recognize and reward these shared upstream contributions. The familiar language of “dual use” gestures toward this reality by acknowledging that a single technology can serve more than one end. But it remains a half-measure. By framing multiplicity as a small set of predefined applications (typically civilian vs. military), it still treats translation as movement along a single lane from research to a designated use. The harder truth is that the path from basic research to market is not one roadmap but a many-to-many mapping: a single upstream capability seeds applications across unrelated sectors, and any given product draws on capabilities that no one agency funded or foresaw. Policies that credit only a designated downstream use will systematically undervalue the shared upstream capacity on which all of them depend. Translation policy should strengthen the connective tissue between discovery and use—testbeds, standards, manufacturing capacity, regulatory pathways, procurement, and access—while allowing foundational research to be judged by discovery rather than immediate commercial yield. Stronger bridges are the objective; turning every grant into a product pipeline is not.

## Build democratic legitimacy and institutional resilience

The fifth principle concerns the relationship between scientific capability and democratic authority. Emerging technologies are too consequential to be governed only after crises occur, but science is too dependent on intellectual independence to be directed by short-term political preference. A renewed compact must therefore combine anticipatory governance, scientific integrity, public accountability, and resilience through political change. These are not hypothetical risks: since early 2025, hundreds of active NSF awards have been terminated, NIH grant cancelations have been ruled “void” and “illegal” in federal court, and independent indices now record a decline in US academic freedom (51, 52); a reminder that resilience is the precondition for every other principle, not an afterthought. Anticipatory governance begins before deployment. AI, autonomous laboratories, advanced biotechnology, quantum technologies, and new materials can alter labor markets, health systems, security practices, education, and the evidentiary basis of public policy. The Organisation for Economic Co-operation and Development has framed anticipatory governance around guiding values, strategic intelligence, stakeholder engagement, agile regulation, and international cooperation (53). The responsible research and innovation tradition similarly emphasizes anticipation, inclusion, responsiveness, and increasing levels of institutional ambition (54). These approaches will matter only if they move beyond principles. The proliferation of AI ethics statements has shown how readily public commitments can exceed incentives for enforcement. Chesterman describes this gap as a tragedy of AI governance: actors benefit from announcing principles even when no institution has the authority or motivation to constrain deployment (55). For the research enterprise, the lesson is that governance cannot be an ethics code appended to an otherwise unchanged program. It must be incorporated into program design, evaluation, auditing, standards, and meaningful avenues for public recourse. Useful precedents already exist. The Human Genome Project’s Ethical, Legal, and Social Implications program embedded ethical and social inquiry within scientific development rather than adding it after the fact, while Australia’s gene-technology regulator combines independent technical assessment, ethics and community expertise, and structured public consultation within a dedicated regulatory institution (56, 57). These models suggest that anticipatory governance requires durable institutional homes, dedicated resources, and formal authority—not merely voluntary principles. Resilience is the institutional counterpart of anticipation. Advisory bodies, scientific-integrity protections, long-term programs, and research infrastructures should be capable of surviving changes in administration without becoming immune to democratic scrutiny. The aim is neither scientific autonomy without accountability nor political control of scientific judgment. It is independence in the assessment of evidence combined with accountability for institutional performance, public value, integrity, and equitable access. This balance requires a civic constituency for science. Periodic public communication campaigns cannot substitute for sustained relationships among researchers, communities, schools, elected officials, professional societies, laboratories, industry, and public institutions. Trust grows when institutions demonstrate competence, acknowledge uncertainty and error, distribute benefits broadly, and create meaningful routes for participation. The public should not be asked to defend science as an abstract cultural good. A substantial body of research on psychological distance and message framing bears this out: abstract, distant appeals engage the public far less than concrete, proximate ones, and vivid, tangible information shapes judgments more powerfully than abstract summaries (58, 59). It should instead be able to see how the capacity for discovery strengthens health, prosperity, safety, freedom, and preparedness. A policy meets this criterion when it embeds anticipatory review, enforceable scientific-integrity protections, meaningful public participation, and continuity across political transitions. Ethics statements without enforcement, political control of scientific judgment, and insulation from democratic scrutiny all miss the target.

## From a US compact to an international dialog

This renewed compact begins as an American proposition because the institutional lineage it revisits originated in the United States. The postwar American research system also shaped scientific institutions well beyond its borders. But the problems that now motivate renewal are not exclusively American. Many countries face versions of the same tensions: openness vs. security; scientific autonomy vs. democratic accountability; global mobility vs. national competition; technological speed vs. regulatory capacity; research excellence vs. geographic and social inclusion; and innovation vs. public legitimacy. The institutional answers will differ across political systems, funding structures, and histories. A renewed compact should not become a universal template exported without context. Nevertheless, scientific knowledge, talent, risks, and public challenges cross borders even when governance remains primarily national. Pandemics, climate and energy systems, AI, supply chains, cybersecurity, and the integrity of the global knowledge base cannot be managed through national capacity alone. The compact should therefore include trusted international collaboration, shared standards, reciprocal talent exchange, scientific diplomacy, and governance mechanisms capable of operating across jurisdictions. The international dimension also disciplines the domestic argument. National scientific leadership should not be defined as the accumulation of knowledge at the expense of others. Durable leadership comes from building institutions that others trust, attracting talent without coercion, producing knowledge that can be scrutinized, and governing powerful technologies in ways compatible with democratic values and human flourishing. A new compact should strengthen national capability while enlarging the global conditions for responsible discovery. The United States could initiate or even lead this conversation, but it should do so as a partner. Discovery is not a territory to be possessed. It is shared infrastructure: a horizon of questions, capabilities, and responsibilities held in common by and for humanity.

## Renewal by design

The postwar compact was transformative because it linked scientific freedom to public responsibility and translated long-term investment into national capability. Its institutions remain essential, but they were not designed for the conditions now confronting them: accelerating and convergent fields, globally mobile talent, administrative saturation, geopolitical pressure, rapidly emerging technologies, polarized trust, and the possibility of abrupt institutional disruption. The answer is neither to abandon the system nor to rebuild it exactly as it was. The Discovery Compact sets out five principles for revival. They are criteria against which programs, institutions, and reforms should be judged. (i) Do they sustain discovery as infrastructure? (ii) Do they move at the speed of science without abandoning rigor? (iii) Do they cultivate people, openness, and mobility? (iv) Do they translate knowledge without consuming the source of discovery? (v) Do they create democratic legitimacy and institutions capable of surviving political change? Developing the compact will require a process as important as the resulting policies. Interdisciplinary training that connects technical work with the humanities, social sciences, and law should be treated as part of scientific capability rather than as an optional supplement. Early- and mid-career researchers, including those considering leaving or recently pushed out of the research enterprise, should be treated as sources of strategic insight: participants in advisory structures, roadmapping exercises, program design, and evaluations of how policies operate in practice. International partners must be involved from the outset in shaping the norms, institutions, and collaborations of a shared scientific future. We have an opportunity to claim the future by deliberately redesigning the discovery enterprise rather than allowing its foundations to erode by default. The work of discovery is never finished, but the institutions that allow free societies to pursue it are not self-renewing. They must be rebuilt deliberately for every new age.
